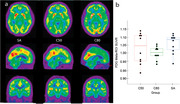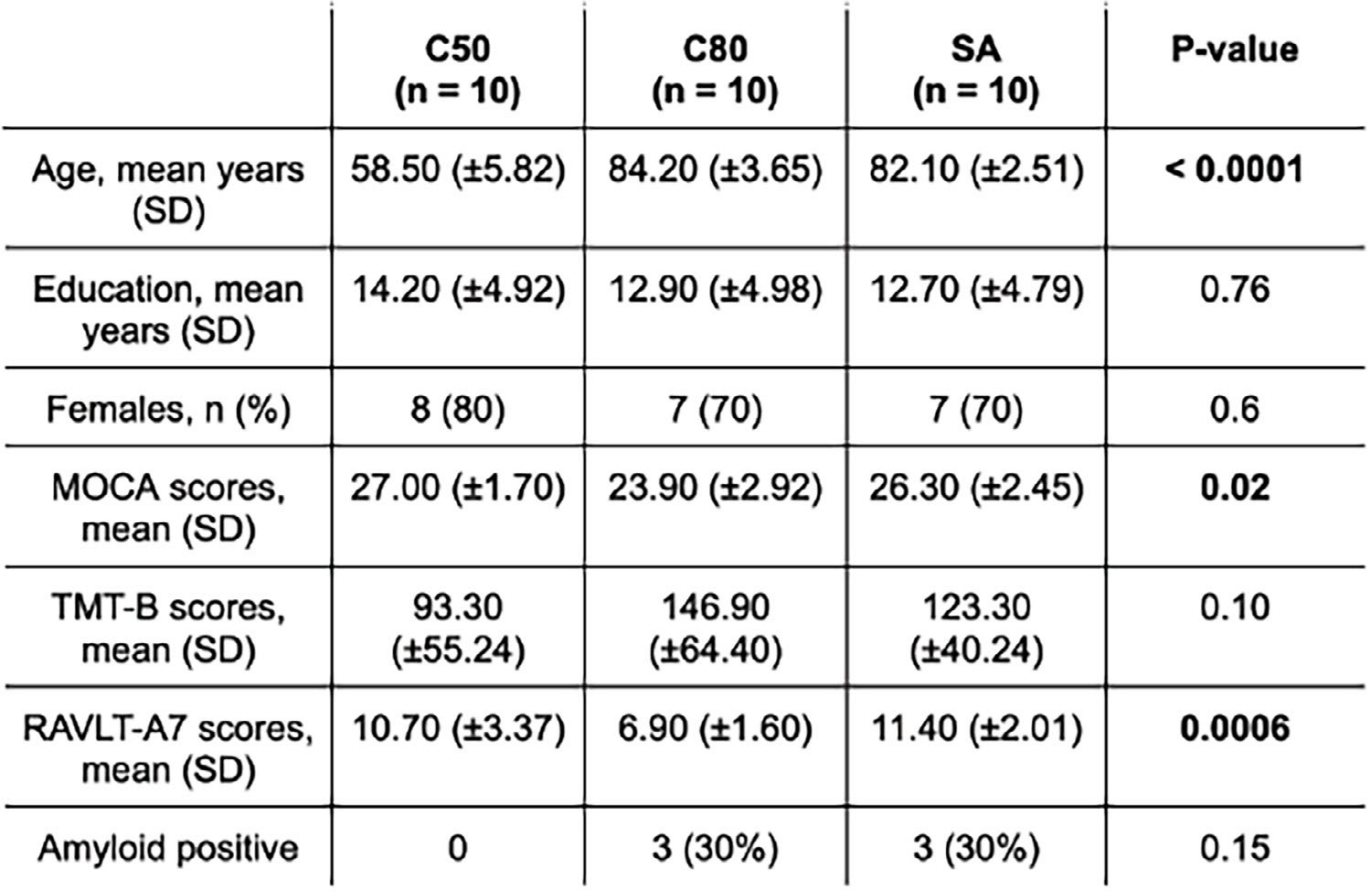# Neural underpinnings of cognitive resilience in Brazilian SuperAgers

**DOI:** 10.1002/alz.088159

**Published:** 2025-01-09

**Authors:** Wyllians Vendramini Borelli, Eduardo Leal‐Conceicao, Lucas Porcello Schilling, Mirna Wetters Portuguez, Eduardo R. Zimmer, Jaderson Costa da Costa

**Affiliations:** ^1^ Universidade Federal do Rio Grande do Sul, Porto Alegre, Rio Grande do Sul Brazil; ^2^ INSCER, Porto Alegre, RS Brazil; ^3^ Brain Institute of Rio Grande do Sul, PUCRS, Porto Alegre, RS Brazil; ^4^ Graduate Program in Biomedical Gerontology, School of Medicine, PUCRS, Porto Alegre Brazil; ^5^ McGill University, Montreal, QC Canada; ^6^ Brain Institute of Rio Grande do Sul ‐ Pontifícia Universidade Católica do Rio Grande do Sul, Porto Alegre, Rio Grande do Sul Brazil

## Abstract

**Background:**

Although cognitive decline is a trait related to aging, some individuals are resilient to the aging process, defined as SuperAgers. Studying the neural underpinnings of SuperAgers may improve the understanding of AD pathology. In this study, our aim was to analyze amyloid and neurodegeneration imaging biomarkers in SuperAgers.

**Method:**

We recruited 228 Brazilian adults and older adults to participate in this study. They underwent a neuropsychological battery, followed by PET [11C] PIB and [18F]FDG imaging. Individuals were classified as SuperAgers when at 80 years of age or above, episodic memory scores similar to 50‐65 normative data, and within 1 SD of normative values for age and education for executive functions, fluency and naming. Amyloid load and glucose metabolism standardized uptake value ratio (SUVR) was calculated using cerebellar crus and whole brain, respectively. SUVR values from predefined AD meta‐ROI for PIB (prefrontal, orbitofrontal, parietal, temporal, anterior and posterior cingulate and precuneus) and FDG (angular gyrus, posterior cingulate, and inferior temporal cortical) were extracted. No covariates were included in the models since sex and education were paired.

**Result:**

After initial screening, a total of 10 young controls (C50), 10 age‐matched controls (C80) and 10 SuperAgers (Table) SA, mean age 82.1±2.51 years) were included. Educational levels were similar between groups (p>0.05), while episodic memory scores were similar only between SA (10.7±3.4) and C50 (11.4±2) but higher than C80 (10.5±3.4, p<0.001). Amyloid SUVR was lower in C50 than older groups, but similar between SA and C80 (SA: 1.25±0.2, C80: 1.32±0.3, C50: 1.07±0.1, p = 0.03). FDG SUVR was increased in SA (Figure) when compared with C80 (1.06±0.1 vs. 1.01±0.1, p = 0.02), but similar to C50 (1.03±0.1, p = 0.21).

**Conclusion:**

Our findings indicate that SuperAgers are more resilient to amyloid burden and seem to cope with AD pathology by increasing brain glucose metabolism. The higher FDG metabolism seen in SuperAgers compared with age‐matched controls may reflect a compensatory biological response, which ultimately leads to cognitive resilience.